# Genetic requirements and transcriptomics of *Helicobacter pylori* biofilm formation on abiotic and biotic surfaces

**DOI:** 10.1038/s41522-020-00167-3

**Published:** 2020-11-27

**Authors:** Skander Hathroubi, Shuai Hu, Karen M. Ottemann

**Affiliations:** 1 Department of Microbiology and Environmental Toxicology, University of California, Santa Cruz, CA 95060 USA; 2 Present Address: Institüt für Biologie/Mikrobiologie, Humboldt-Universität zu Berlin, 10555 Berlin, Germany

**Keywords:** Biofilms, Microbial genetics

## Abstract

Biofilm growth is a widespread mechanism that protects bacteria against harsh environments, antimicrobials, and immune responses. These types of conditions challenge chronic colonizers such as *Helicobacter pylori* but it is not fully understood how *H. pylori* biofilm growth is defined and its impact on *H. pylori* survival. To provide insights into *H. pylori* biofilm growth properties, we characterized biofilm formation on abiotic and biotic surfaces, identified genes required for biofilm formation, and defined the biofilm-associated gene expression of the laboratory model *H. pylori* strain G27. We report that *H. pylori* G27 forms biofilms with a high biomass and complex flagella-filled 3D structures on both plastic and gastric epithelial cells. Using a screen for biofilm-defective mutants and transcriptomics, we discovered that biofilm cells demonstrated lower transcripts for TCA cycle enzymes but higher ones for flagellar formation, two type four secretion systems, hydrogenase, and acetone metabolism. We confirmed that biofilm formation requires flagella, hydrogenase, and acetone metabolism on both abiotic and biotic surfaces. Altogether, these data suggest that *H. pylori* is capable of adjusting its phenotype when grown as biofilm, changing its metabolism, and re-shaping flagella, typically locomotion organelles, into adhesive structures.

## Introduction

*Helicobacter pylori* is a significant human pathogen that is difficult to cure. *H. pylori* infects more than half of the world’s population, making it one of the most common bacterial infections^1^. *H. pylori* causes chronic gastritis, duodenal and gastric ulcers, and significantly increases the risk of gastric adenocarcinoma and mucosal-associated-lymphoid type (MALT) lymphoma. *H. pylori* disease is significant: Gastric cancers kill over 700,000 people per year, and peptic ulcer disease has an estimated cost of six billion dollars in the United States alone^1,2^. Many of these diseases could be prevented by curing the underlying *H. pylori* infection^1,3^.

Antibiotics are used to cure *H. pylori* infections, but successful treatment remains challenging. The standard of care is a 2-week course of a combination of a proton pump inhibitor and two-three antibiotics (clarithromycin, metronidazole, amoxicillin, or tetracycline), but this treatment leaves ~25% of people uncured^4,5^. It is not yet fully understood why cure is so difficult, because *H. pylori* strains are not typically multidrug resistant. Rising resistance to single antibiotics, especially clarithromycin and metronidazole, is a growing concern and certainly accounts for some treatment failure^5,6^. An additional explanation may be that some *H. pylori* are conditionally antibiotic tolerant due to their growth state and concomitant gene expression. One growth state associated with antibiotic tolerance is biofilm growth^7–10^. *H. pylori* can grow as biofilm in vitro^11–16^ and in vivo^17–19^. Recent studies have suggested that biofilm-grown *H. pylori* are tolerant to the commonly used antibiotics clarithromycin, amoxicillin, and metronidazole, and express high levels of putative antibiotic efflux pumps^20–22^. These findings suggest that biofilm formation could be a contributor to *H. pylori* persistence and the difficulty in curing this infection.

Studies on *H. pylori* biofilms have been relatively few, but recent contributions from high throughput ‘omics’ strategies such as genomics, transcriptomics, and proteomics have identified several biofilm properties^16,23–25^. Indeed, *H. pylori* biofilm are less metabolically active than planktonic cells, possess low protein translation, and display multiple aspects of stress responses^16^. Biofilms have been shown to rely on membrane proteins, genes products of the cytotoxin-associated gene pathogenicity island (*cag*PAI), which express a type IV secretion system (*cag*-T4SS)^23,24^, and flagellar genes^16^. Flagellar filaments have been shown to be part of the *H. pylori* biofilm matrix along with proteins, eDNA, carbohydrates, and LPS^16,25–27^.

Nevertheless, *H. pylori* biofilm studies have been slowed in part by the fact that *H. pylori* strains are highly variable. Several lab strains have become model systems in general, however, due to their properties such as ease of growth, solid genetics, or ability to reliably infect mice. One of these is *H. pylori* strain G27. *H. pylori* G27 is known for its relatively straightforward genetics and reliable growth. Several recent papers have used *H. pylori* G27 to document important aspects of *H. pylori* biofilm formation. These insights include that G27 forms biofilms on many abiotic and biotic surfaces under standard lab conditions, has a matrix composed mainly of proteins but also some eDNA and carbohydrates, that conditions such as lowered Zn- and Mn- promote biofilm formation in part due to LPS modifications, and that AI-2 quorum sensing and the ArsRS regulatory system are important players^14,26,28–30^. However, the genetic pathways for biofilm formation have not yet been characterized in any *H. pylori* strain, including G27. As the next logical step in characterizing *H. pylori* biofilm formation, we undertook studies to further *H. pylori* G27 as a model system. Here, we characterize *H. pylori* G27’s biofilm formation on abiotic and biotic surfaces, and perform genomic and transcriptomic approaches to unravel genes associated with the biofilm mode of growth in this strong biofilm-forming *H. pylori*.

## Results

### *H. pylori* strain G27 forms large-mass biofilms under standard laboratory conditions

**Fig. 1:**
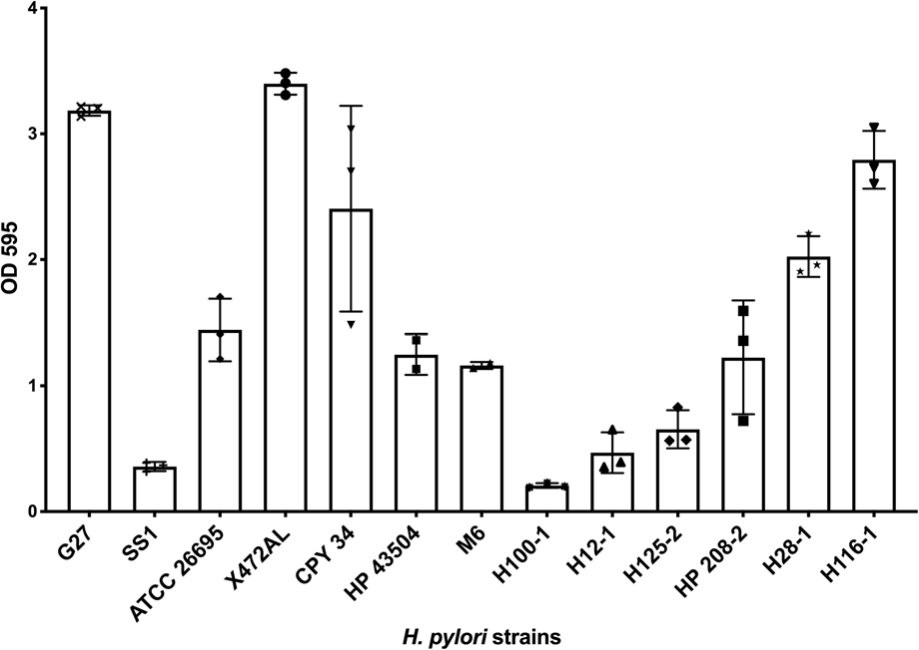
Specific *H. pylori* strains form biofilms in rich media. Biofilm formation of *H. pylori* strains was assessed using the microtiter plate crystal violet biofilm assay. Strains were grown for three days in BB10, with no shaking, under 10% CO_2_, 5% O_2_ and 85% N_2_. Results represent the crystal violet absorbance at 595 nm, which reflects the biofilm biomass. Experiments were performed three independent times with at least six technical replicates for each. Error bars represent standard error of the mean.

Multiple *H. pylori* strains form biofilms, including the *H. pylori* model strain G27^12,29–32^. For the most part, these studies have used various media and conditions, and not compared the strains side-by-side. Therefore, we first compared the biofilm-forming ability of strain G27 with several *H. pylori* reference and clinical strains. We used the microtiter plate biofilm assay, referred to as abiotic, with 3 days of growth in Brucella Broth with 10% heat-inactivated fetal bovine serum (BB10), no antibiotics, and microaerobic conditions (10% CO_2_, 5% O_2_, 85% N_2_). After incubation, adherent cells were washed and stained with crystal violet for quantification. All strains adhered to the plastic surfaces at the bottom of the well, and formed crystal-violet stained biomass (Fig. 1). Some strains, including G27, also formed biofilms at the air–liquid interface as rings on the plastic and/or a thin, fragile, floating pellicle, as previously noted^28^. Quantification of the adherent biofilms showed that some strains displayed a large amount of biofilm mass, and others displayed less (Fig. 1). Lab strains G27, X47AL, and CPY3401 all formed high amounts of biofilm, while others formed low amounts, including the common mouse-infecting *H. pylori* strain SS1. Varied biofilm biomass was also displayed by clinical isolates, suggesting this is a strain-specific property and not associated with lab culture (Fig. 1). Time course analysis suggested that biofilm mass was detectable at 24 h, and steadily increased to a maximum at 3 days of growth (Supplementary Fig. 1A and ref. ^26^). Biofilm formation was influenced by some environmental parameters including pH: the biomass decreased as the pH was lowered from neutral to pH 3 (Supplementary Fig. 1B). Overall, these experiments show that *H. pylori* G27 is a type of *H. pylori* strain that forms a high level of biofilm biomass under lab conditions, with three days of lab culture resulting in maximum biofilm formation.

### *H. pylori* G27 biofilm consists of three-dimensional (3D) aggregates of multiple cell layers with flagella filaments and pili-like structures

**Fig. 2:**
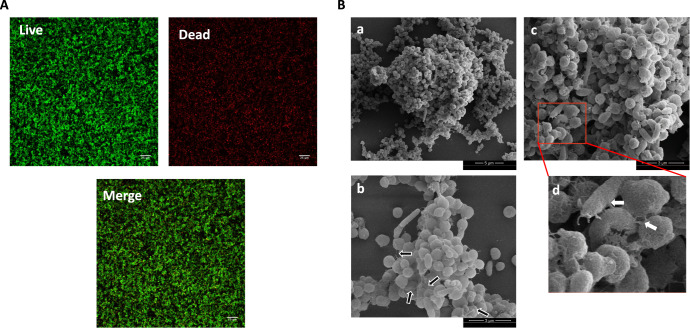
*H. pylori* G27 biofilm characterization. *H. pylor*i G27 was allowed to form biofilms for three days on plastic as in Fig. 1. **A** Confocal scanning laser microscopy (CSLM) images (top view) of 3-day old *H. pylori* G27 biofilms stained with LIVE (green)/DEAD (red) stain. **B** SEM images show cell-to-cell interactions through flagellar filaments and pili-like structures. High magnification in d is derived from the red-boxed area of c. Black arrows indicate flagella filaments and white arrows indicate the presence of pili-like structures connecting cells togethe. SEM micrographs of *H. pylori* biofilm at 5000x (a) and 12500x (b and c).

We next asked about the architecture of the *H. pylori* G27 abiotic surface-attached biofilms. Previous work had shown that G27 and other *H. pylori* have a biofilm matrix consisting mostly of proteins along with some eDNA^26^. Confocal laser-scanning analysis of *H. pylori* G27 biofilms showed multiple layers of cells with a thickness of 15–20 μm^22^. At 3 days of growth, there were approximately equal proportions of live (green; SYTO 9-stained cells) and dead or damaged cells (red; Propidium iodide-stained cells) that were spread throughout the biofilm (Fig. 2A). Quantification of the percent of each population yielded a live/dead ratio of 1.17 ± 0.14.

Scanning electron microscopy (SEM) further showed that *H. pylori* G27 biofilms consisted of 3D structures composed of aggregated bacteria attached to the surface and to each other (Fig. 2B). These aggregates contained predominant coccoid forms along with some rod-shaped bacilli (Fig. 2B). Previous reports have shown that 72 h of growth in static condition, as used here, results in both planktonic and biofilm cells becoming predominantly coccoid^16^. The rod-shaped cells were mostly characterized by straight-rod morphology of about 2–3 μm long by 0.5 μm wide, whereas the coccoid cells were smaller, with a diameter around 0.4–0.6 μm (Fig. 2B). As reported previously, the *H. pylori* G27 biofilms contained flagellar filaments, based on size, appearance, and polar location^16^. We also observed pili-like structures at cell-to-cell contacts, however, their nature and role remain unknown (Fig. 2B). Overall, these analyses show that *H. pylori* G27 biofilms consist of layers of adherent coccoid and rod-shaped cells, as well as flagella and pili-like structures.

### *H. pylori* G27 forms biofilm on AGS gastric epithelial cells

**Fig. 3:**
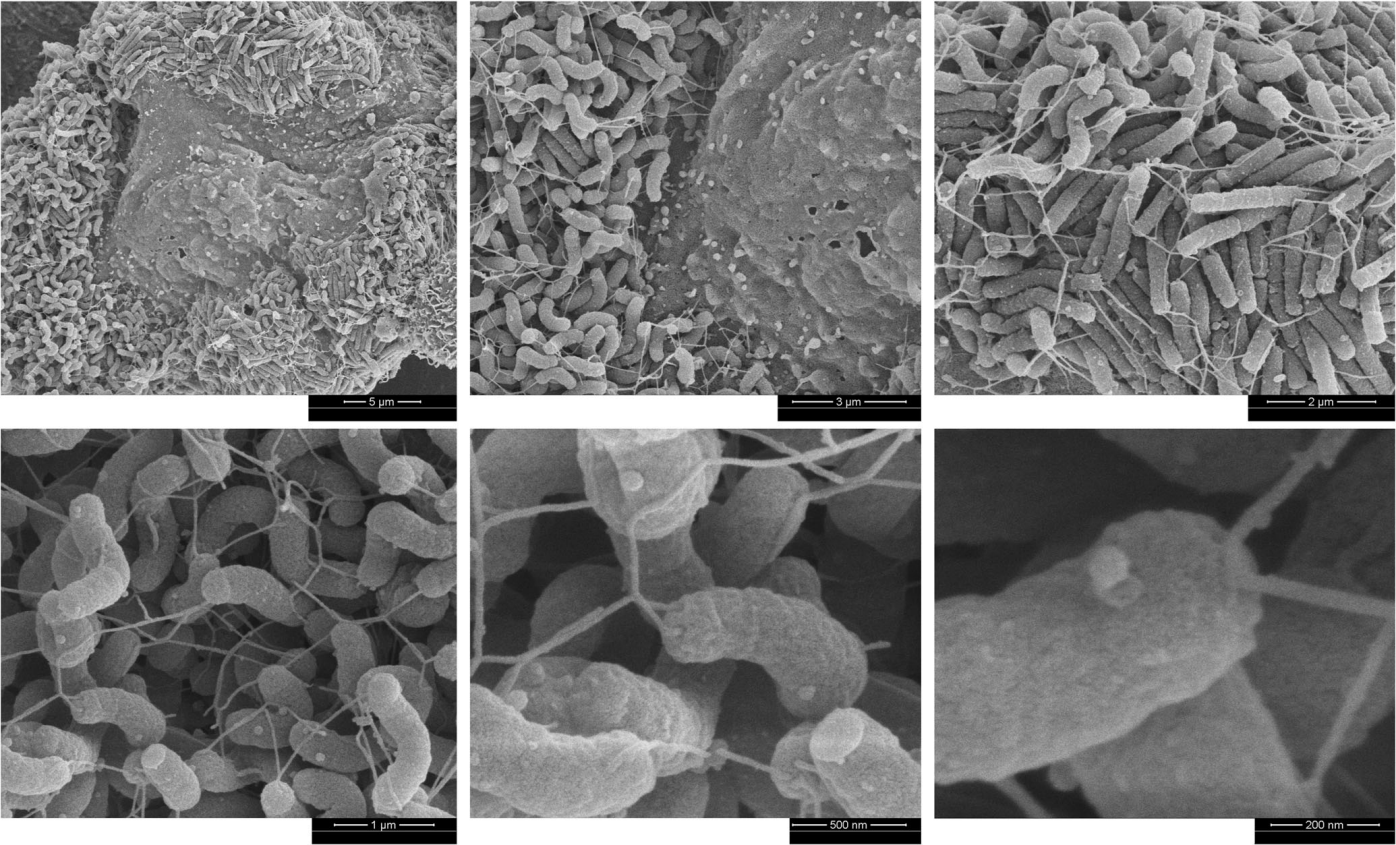
*H. pylori* G27 biofilms formed on AGS cells. SEM images showing 3-day old *H. pylori* G27 wild-type (WT) biofilms formed on AGS cells. Magnified images highlight bacteria-to-bacteria and bacteria-to-cell interactions through flagella.

We next examined biofilm formation on gastric epithelial cells as a substrate to better mimic aspects of the in vivo environment, recognizing that using plastic or glass as a substrate might not reflect the biofilm formed by *H. pylori* during stomach colonization. Previous studies had reported that *H. pylori* forms microcolonies in vitro on the surface of epithelial cells and in vivo on the mucosal surface and in the glands^29,33–37^, but whether these were biofilms was not clear. To examine *H. pylori* growth on epithelial cells in more detail, we used the *H. pylori* model human gastric epithelial adenocarcinoma cell line AGS (ATCC CRL 1739). *H. pylori* G27 was co-incubated with confluent AGS cells for 72 h, the length of time it took for abiotic biofilms to form a dense structure. After this time, the bacterial growth pattern was analyzed by SEM. After 72 h of growth, *H. pylori* was found *H. pylori* exclusively in large 3D confluent bacterial masses on the cell surface (Fig. 3). The close multi-layered bacterial packing and connectivity was similar in appearance to the in vitro biofilms. Within these aggregates, an extensive mesh of flagellar filaments could be seen connecting bacteria to each other and to AGS cells (Fig. 3), similar to the appearance of flagella in the abiotic biofilms^16,38^. We refer to these bacterial aggregates as biotic biofilms, given to the 3-dimensional structures formed and the filamentous mesh of flagella that seems to contribute to adhesion and cohesion.

The biotic biofilm *H. pylori* were composed almost exclusively of spiral and rod-shaped cells, as compared to ones in the abiotic biofilms in which bacteria were mostly coccoid (Fig. 2B). We also noted the *H. pylori* aggregates were not homogeneously distributed over the surface of the AGS cells but found largely in the recessed cell-cell junction areas (Fig. 3), as reported previously^33^.

### *H. pylori* biotic biofilms require flagella for wild-type formation

To further investigate the importance of flagella in initial attachment and biofilm formation on AGS cells, we tested biotic biofilm formation of mutants that were either (1) flagellated but non-motile (Fla^***+***^ Mot^***−***^) (*motB*) or (2) aflagellated and non-motile ((Fla^***−***^ Mot^***−***^) (*fliA*). Equal numbers of these *H. pylori* strains were co-incubated with AGS cells as above. We monitored both simple attachment after 1 h of incubation and biofilm formation after 72 h of incubation. In both cases, we quantified cells by CFU plating as done previously^39–41^. This approach only monitors the live bacteria, but given the abundance of spiral bacteria in the biotic biofilm we felt this method would capture biofilm formation.

**Fig. 4:**
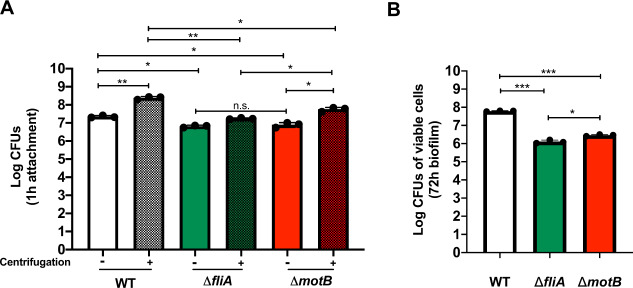
*H. pylori* flagella is essential for initial attachment and biofilm on AGS cells. Attachment and biofilm formation of *H. pylori* G27 wild type (WT), mutant *fliA* (aflagellated and non-motile), and mutant *motB* (flagellated and non-motile) was analyzed by counting colony-forming unit (CFU/ml). **A** *H. pylori* WT and mutants initial attachment on AGS cells were measured after 1 h infection, with (solid bars) or without (striped bars) centrifugation. **B** *H. pylori* cells on the surface were measured after 72 h to monitor biofilm formation using CFUs. Experiments were performed three independent times with at least three technical replicates for each. Error bars represent standard error of the mean. Statistical analyses were performed using one-way ANOVA with Tukey post hoc test (*P < 0.01; **P < 0.001; ***P < 0.0001; n.s., no significance).

When we compared wild-type *H. pylori* G27 to its Fla^***+***^ or Fla^***−***^ mutants after 1 h of incubation, we found that both types of mutants had significant attachment defects (Fig. 4A). Since motility is well known to be relevant to colonization^42^, we applied an additional centrifugation step to evaluate flagella versus motility in adherence (Fig. 4A). As expected, centrifugation greatly enhanced the number of *H. pylori* attached to the cells: Without centrifugation, about 3 × 10^7^ wild-type *H. pylori* attached to the surface of AGS cells in a 1 h incubation period, and about 10-fold more *H. pylori* were attached when centrifugation was applied (Fig. 4A). When we compared wild-type *H. pylori* G27 to its Fla^***+***^ or Fla^***−***^ mutants upon centrifugation, we found that both types of mutants had significant attachment defects, and they were not different from each other (Fig. 4A). However, the attachment of the flagellated Fla^***+***^ *motB* mutant did not match wild-type levels (Fig. 4A), indicating that flagella rotation or corresponding movement is involved in the initial attachment process as well.

To analyze biofilm formation, we analyzed non-centrifuged cells for longer incubations (72 h post infection). We used non-centrifuged cells because there was not a significant difference between Fla+ and Fla- variants in early attachment, and thus we reasoned this approach would allow us to assess whether flagella were important for biotic surface biofilm formation. Both mutants colonized AGS cells at lower numbers than wild-type strains, and the flagellated Fla^+^ *motB* mutant exhibited a significantly higher colonization than the aflagellated Fla^−^ *fliA* mutant (Fig. 4B). Taken together, these results show that *H. pylori* employs flagella for adherence and biofilm formation on AGS cells, similar to what has been reported for abiotic surfaces.

### Identification and characterization of genes required for biofilm formation

**Fig. 5:**
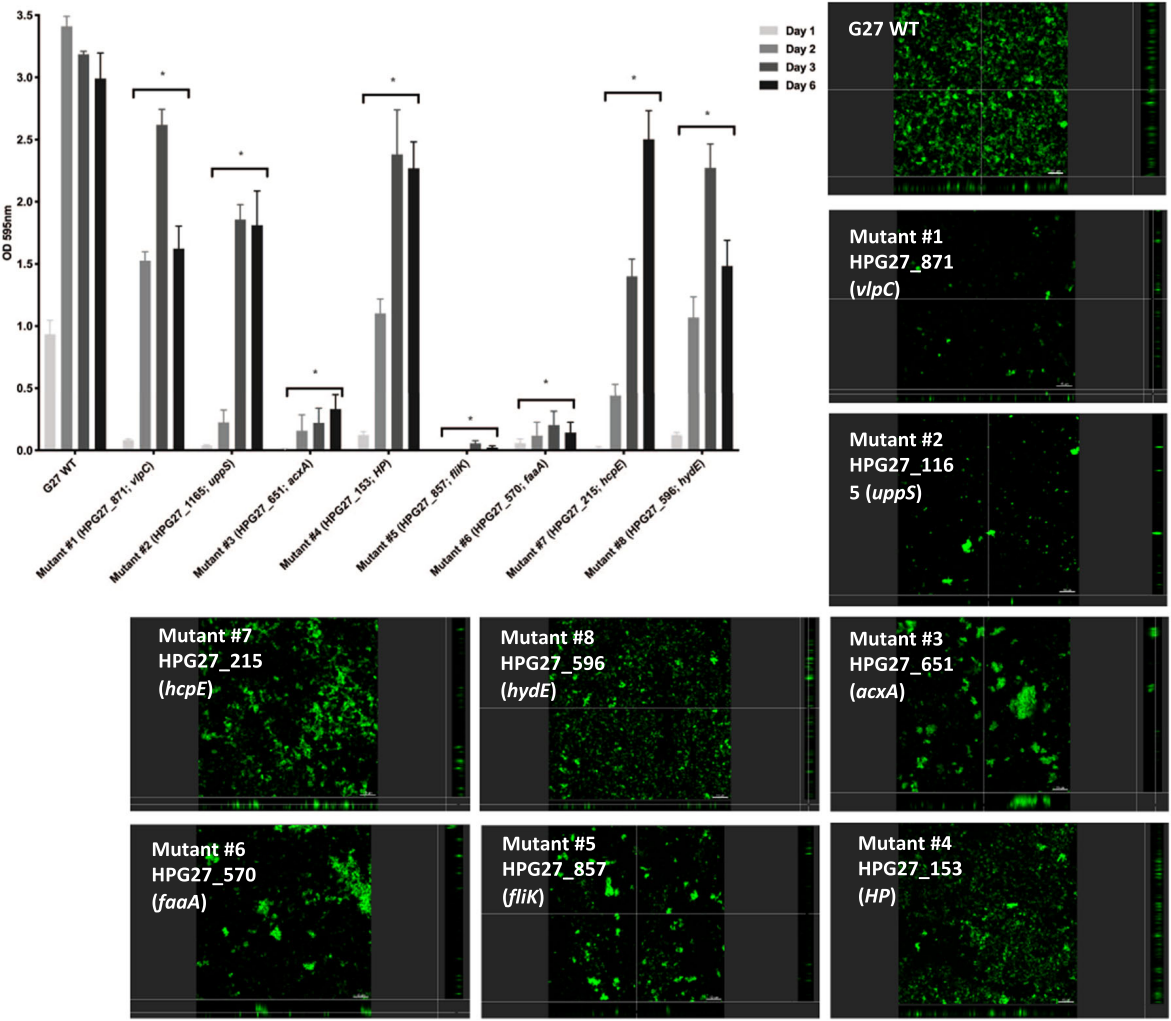
Biofilm formation by *H. pylori* G27 WT and biofilm-defective mutants. Biofilm formation was assessed using the microtiter plate biofilm assay and confocal laser-scanning microscopy over three days. Results represent the crystal violet absorbance at 595 nm, which reflect the biofilm biomass. Experiments were performed three independent times with at least three technical replicates for each. Error bars represent standard errors for each average value. CLSM micrographs compare biofilm formation of the wild-type G27 strain and biofilm-defective mutants. Biofilms were stained with FM 1–43, which become fluorescent once inserted in the cell membrane. HP hypothetical protein, WT Wild-type. Statistical analyses were performed using ANOVA (*P < 0.05).

Our data above support that the *H. pylori* strain G27 forms biofilms on both abiotic and biotic surfaces. However, the genes required for *H. pylori* biofilm are still largely unknown. We thus performed a genetic screen to find genes required for *H. pylori* biofilm formation, using an *H. pylori Tn*-7-based transposon-based mutant library^43^. The *H. pylori* G27 *Tn*-7 mutant pool forms biofilms on polystyrene plates, and we thus screened for mutants unable to form biofilms by repeatedly collecting the supernatant, which should contain potential biofilm-defective mutants. This biofilm growth/supernatant collection approach was repeated multiple times. At the end of the screening period, supernatant cells were plated and 97 colonies were randomly picked and assessed for biofilm formation using crystal violet staining. Eight of these displayed significant biofilm formation defects (Fig. 5), while 89 displayed wild-type biofilm formation. To ensure that the biofilm defects were not associated with growth defects, we measured the growth rates and compared them to that of the wild-type strain (Supplementary Fig. 2). Of the eight mutants, all were like wild type except mutant #6, which had a substantially decreased growth rate (Supplementary Fig. 2).

We next asked whether the eight biofilm-defective mutants had a complete or a partial loss of biofilm formation, by carrying out a time course 96-well plate crystal violet assay (Fig. 5). Some mutants (numbers 3, 5, 6) were completely unable to form biofilms, while others were able to form some level of biofilm that did increase over time (1, 2, 4, 7, and 8) (Fig. 5). These data suggest the mutants can be grouped into two classes: total or partial biofilm formation defect.

To further confirm the biofilm defects, these mutants were analyzed by CSLM after 3- days of growth. Biofilm-defective mutants all had visible defects in biofilm formation compared to wild-type parental strain G27 (Fig. 5). The mutants with the strongest defect in the crystal violet plate assay, mutants #3, #5, and #6, seemed to form only microcolonies without further development as demonstrated by severe reduction in biofilm biomass (Fig. 5). Mutant strains with partial crystal violet defects, e.g., mutant #7 and #8, were able to spread better across the plate surface, but still not able to form substantial biofilms compared to wild type (Fig. 5). Interestingly mutants #1 and #2 seemed to be more defective in CSLM compared to the 96-well crystal violet assay. This difference may be due to the use of a glass surface for CSLM versus a plastic one for the screening assay, suggesting potentially interesting differences in surface interactions. Overall, the CSLM analysis supports that our identified mutants have defects in biofilm formation.

We next mapped the location of the biofilm-defective transposon mutants, using nested PCR and a combination of transposon-specific and random primers to create PCR products for each transposition event. Sequencing of these PCR products identified eight non-redundant disrupted genes (Table 1 and Supplementary Fig. 3). Mutants with severe biofilm defects mapped to genes required for acetone metabolism (*acxA*/HPG27_651, mutant #3) and flagellar motility (*fliK*/HPG27_857, mutant #5 and *faaA*/HPG27_570 mutant #6). *acxA* encodes the alpha subunit of acetone carboxylase, an enzyme that converts acetone to acetoacetate and is required in some microbes for growth on acetone as a carbon and energy source^44^. *fliK* encodes the flagella hook-length control protein, while *faaA* encodes a flagella-associated autotransporter. Mutants lacking *fliK* are aflagellated and non-motile^45^ while those lacking *faaA* have decreased flagellation and motility^46^. The *faaA* mutant had a growth defect (Supplementary Fig. 2), which might contribute to its inability to form biofilms.

**Table 1:** *H. pylori* G27 reduced biofilm mutants.

Mutant ID	G27 (26695) locus tag	*Tn* location (bp)#	Gene product	Description
#1	HPG27_871 (HP0922)	952330	VlpC	Large outer membrane autotransporter protein; unknown function.
#2	HPG27_1165 (HP1221)	1284748	UppS	Putative undecaprenyl pyrophosphate synthase; possible role in peptidoglycan synthesis.
#3	HPG27_651^a^ (HP0695)	712390	AcxA	Acetone carboxylase alpha subunit; acetone utilization.
#4	HPG27_153 (HP0168)	171244	87 amino acid hypothetical protein	Unknown function, no conserved domains.
#5	HPG27_857 (HP0906)	922300	FliK	Flagellar hook-length control protein; motility.
#6	HPG27_570 (HP609-619)	622795	FaaA	Flagella-associated autotransporter, outer membrane; Motility.
#7	HPG27_215 (HP0235)	241131	HcpE	Putative beta lactamase. cellular defense; antibiotic resistance.
#8	HPG27_596 (HP0635)	650623	HydE	Protein involved in localizing hydrogenase complex to the membrane; hydrogen metabolism.

Transposon (Tn) location is given using the numbering of the G27 genome (31).

^a^HPG27_651 was originally not annotated on the genome.

Partially defective biofilm mutants mapped to genes for an outer membrane autotransporter protein of unknown function (*vlpC*. mutant 1)^46^, peptidoglycan synthesis and beta lactam resistance (*uppS*, mutant 2; *hcpE*, mutant 7)^47^, hydrogenase activity (*hydE*, mutant 8)^48^, and a small gene of unknown function (mutant 4). While no clear pattern emerged from these mutants, the data are consistent with the idea that multiple processes are important for robust biofilm formation.

### Transcriptomic analysis reveals multiple genes are associated with biofilm growth including those required for flagella and acetone metabolism

**Fig. 6:**
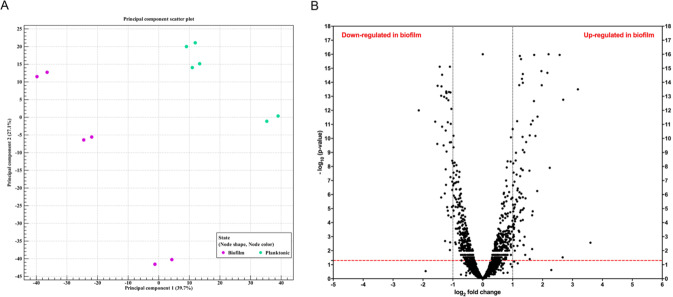
Differential gene expression in *H. pylori* biofilm-grown cells as opposed to planktonic grown cells. *H. pylori* strain G27 was grown as a biofilm or planktonic culture for 3 days, after which RNA was collected and sequenced. **A** Principal component analysis (PCA) of gene expression obtained by RNA-seq between biofilm growing cells and planktonic ones, with three biological replicates and two technical replicates each for *n* = 6. **B** Volcano plot of gene expression data. The *y*-axis is the negative log10 of *P*-values (a higher value indicates greater significance) and the *x*-axis is log2 fold-change or the difference in abundance between two population (positive values represent the upregulated genes in biofilm and negative values represent downregulated genes). The dashed red line shows where *P* = 0.01, with points above the line having *P* < 0.01 and points below the line having *P* > 0.01.

The above work suggests that the *H. pylori* biofilm is a complex structure that likely requires the coordinated expression of many genes. To further *H. pylori* G27 as a model, we identified the major transcriptomic features of its biofilm-grown cells using an RNA-sequencing (RNA-seq). Transcriptomics was recently used to gain important insights about the biofilm growth state of a distinct *H. pylori*, the mouse-infecting strain SS1^16^. For *H. pylori* G27, we had initially hoped to analyze biofilm and planktonic *H. pylori* grown in the same well. This approach was not possible, however, because the G27 strain formed both a dense biofilm on the bottom surface and a thin fragile pellicle at the air–liquid interface that could not be removed and separated from the planktonic cells. This issue led to contamination of the planktonic cells with pellicle ones, so we decided to grow the biofilm and planktonic populations separately. For biofilm growth, cells were grown in static conditions for three days, while planktonic cultures were shaken and grown for 24 h. We chose these time points because in each case the cells had recently entered stationary phase and consisted of largely coccoid non-motile forms, and so we reasoned these conditions would lead to reasonable comparisons. Cells from three biological replicates, with two technical replicates each, were collected and RNA was extracted and sequenced. A total of 9–16 million reads per sample were generated by RNA-seq and mapped to the *H. pylori* G27 complete reference genome^49^. These reads revealed a clustering of the biofilm-grown cells in a distinct population compared to those grown in planktonic conditions (Fig. 6A). The biofilm populations were more variable compared to the planktonic ones, suggesting heterogeneity of biofilm sub-populations. Transcriptomic analysis showed that a total of 136 of 1534 genes (8.86%) were significantly differentially expressed (*P* < 0.05 and log_2_-fold-change >1 or <−1) between biofilm versus planktonic *H. pylori* (Fig. 6B), with 93 genes expressed to significantly higher levels in in the biofilm condition (Table 2 and Supplementary Table 2) and 43 genes significantly downregulated (Table 3 and Supplementary Table 3). To confirm the results obtained by RNA-seq, six differentially expressed genes were quantified by quantitative reverse transcription-PCR (qRT-PCR), and found to display the same expression trend by this method as the original RNA seq (Supplementary Fig. 4). Overall, these results suggest that *H. pylori* G27 grown as a biofilm has a distinct transcription profile.

**Table 2:** List of the top 30 most upregulated genes in *H. pylori* G27 grown in biofilm condition (cutoff ratio ≥1 log2 fold-change and *P*-value < 0.05) using RNA-seq analysis. These genes are all expressed to higher levels in the biofilm cells.

Name	Putative gene product	Fold-change
HPG27_166	Hypothetical protein	12.13
HPG27_979	VirC1	9.07
HPG27_970	VirB11-like	6.43
HPG27_1233	^m6^A methyltransferase	6.35
HPG27_969	Hypothetical protein	5.93
HPG27_1522	Hypothetical protein	4.72
HPG27_270	Chorismate mutase	4.59
HPG27_968	Hypothetical protein	4.46
HPG27_509	Glutamate racemase	3.92
HPG27_1066	^m5^C methyltransferase	3.89
HPG27_324	RluA, pseudouridine synthase D	3.57
HPG27_1059	Hypothetical protein	3.53
HPG27_335	Hypothetical protein	3.39
HPG27_269	LysA, diaminopimelate decarboxylase	3.29
HPG27_671	LptB, ABC transporter	3.26
HPG27_506	CagB, cag island protein B	3.18
HPG27_271	Hypothetical protein	3.17
HPG27_442	^m5^C methyltransferase	3.15
HPG27_834	Hypothetical protein	3.13
HPG27_669	Hypothetical protein	3.06
HPG27_1331	Hypothetical protein	2.97
HPG27_377	TsaB, tRNA A37 threonylcarbamoyladenosine modification protein	2.96
HPG27_72	PrfA, peptide chain release factor RF-1	2.96
HPG27_1241	Thiamine pyrophosphokinase	2.73
HPG27_652	AcxB, acetone carboxylase subunit alpha	2.69
HPG27_589	Hypothetical protein	2.68
HPG27_800	Hypothetical protein	2.63
HPG27_571	ABC transporter, permease	2.62
HPG27_47	HypAV, type II restriction enzyme	2.59
HPG27_1299	HpyAIV, type II restriction endonuclease	2.59
HPG27_660	Hypothetical protein	2.57

**Table 3:** List of the top 30 most downregulated genes in *H. pylori* G27 biofilm conditions compared to cells in planktonic conditions (cutoff ratio ≥ 1 log2 fold-change and *P*-value < 0.05) using RNA-seq analysis, grouped by functional role categories. These genes are all expressed to lower levels in the biofilm cells.

Name	Product	Fold-change
HPG27_256	Ferredoxin	−4.39
HPG27_516	Hypothetical protein	−3.01
HPG27_1015	Zinc-metalloprotease	−2.86
HPG27_1271	Hypothetical protein	−2.84
HPG27_1035	Pseudo uridylate synthase I	−2.71
HPG27_1180	Hypothetical protein	−2.62
HPG27_1008	SodB, iron-dependent superoxide dismutase	−2.61
HPG27_591	Mda66, modulator of drug activity	−2.60
HPG27_527	Hypothetical protein	−2.57
HPG27_937	Hypothetical protein	−2.56
HPG27_338	KgtP, alpha-ketoglutarate permease	−2.47
HPG27_1314	Type III restriction enzyme M protein	−2.45
HPG27_277	AibA, AI-2 periplasmic binding protein	−2.41
HPG27_1376	Hypothetical protein	−2.39
HPG27_1044	Glk, glucokinase	−2.36
HPG27_548	OorD, 2-oxoglutarate oxidoreductase subunit	−2.36
HPG27_1205	NADH-ubiquinone oxidoreductase chain A	−2.35
HPG27_783	TrxA, thioredoxin	−2.33
HPG27_1125	Hypothetical protein	−2.32
HPG27_957	Cyclopropane fatty acid synthase	−2.31
HPG27_1329	Biotin synthetase	−2.31
HPG27_887	Hypothetical protein	−2.31
HPG27_521	Ribosomal protein S21	−2.29
HPG27_1156	Hypothetical protein	−2.25
HPG27_1206	NuoB, NADH oxidoreductase I	−2.25
HPG27_1273	FumC, fumarase	−2.22
HPG27_469	Urease-enhancing factor	−2.21
HPG27_549	OorA, 2-oxoglutarate oxidoreductase subunit	−2.19
HPG27_279	DppC, dipeptide transport system permease protein	−2.14
HPG27_1007	Adhesin-thiol peroxidase	−2.14
HPG27_306	FlgH, flagellar L-ring protein precursor	−2.14

Although there were many genes that differed between the two conditions, we discuss several in this section that we pursued in this study, with an additional few in the discussion. Many of the regulated genes correlated well with previous work about *H. pylori* biofilms, suggesting the *H. pylori* G27 biofilms exemplify typical *H. pylori* biofilms and that our methods were capturing these properties (Tables 2 and 3). Differentially expressed genes included ones with roles in quorum sensing, metabolism, LPS, and flagella. Key genes related to flagella and LPS (*rpoN*, *flaA*, *lptB*) were upregulated, consistent with the known roles of these macromolecules in the *H. pylori* biofilm matrix^16,30^, and agree well with findings from our genetic screen for *fliK* and *faaA* (Table 1). Recently, it has been shown that LPS is modified in the *H. pylori* biofilms^30^, and we observed downregulation of *lpxF*, encoding an enzyme responsible for dephosphorylation of the lipid-A 4′-phosphate group (Supplementary Table 3). Previous studies also showed that AI-2 quorum sensing caused biofilm dispersal^29,50^, and accordingly we saw biofilm cells decrease expression of genes related to AI-2 quorum sensing, including the genes for AI-2 synthesis (*luxS*), an AI-2 periplasmic binding protein (HPG27_277/*aibA)*, and a putative AI-2 exporter (HPG27_526) (Supplementary Table 3).

The transcriptomic results also suggest biofilm cells downregulate some forms of metabolism, including glycolysis, the TCA cycle, and electron transport, while upregulating genes required for hydrogenase, urease, and acetone carboxylase activities (Tables 2 and 3). Several acetone metabolism genes were upregulated in biofilm cells, including *acsA, acxA*, and *acxB*, which encode an acetyl coenzyme A synthetase and acetone carboxylase alpha and beta subunits, respectively (Table 2). The *acxA* gene was also identified in our transposon screen, which reinforces the idea that this gene and/or acetone metabolism might be important for *H. pylori* biofilm formation. Similarly, we detected upregulation of *hypF*, a gene required for producing the Ni-Fe hydrogenase cyanide ligand (45). We also found several genes belonging to T4SS systems (tfs4 and cag-PAI) and genes encoding for restriction-modification (R-M) and DNA repair and protection upregulated in biofilm cells as (Table 2 and Supplementary Table 2). In sum, these results support that our transcriptomic approach has identified key biofilm genes and indicates that *H. pylori* requires LPS, flagella, T4SS and distinct metabolism when grown as biofilm.

### Genes identified through transcriptomics and the mutant screen are required for abiotic biofilm formation

We next sought to evaluate whether genes identified above, in either the transposon mutant approach or as differentially regulated, were important for biofilm formation. We, therefore, selected several genes and generated targeted deletion/insertion mutants in them (Table 1). From the transposon screen, we choose two genes that exemplified either the partial or total biofilm-defective mutants: *hydE*, required for hydrogenase activity, and *acxAB*, coding for the acetone carboxylase. We were particularly interested in these because hydrogenase and acetone metabolism genes were also identified in the transcriptomic approach. From the transcriptomic approach, we chose seven significantly differentially regulated genes that were in the hydrogenase, acetone utilization, or other unknown or in prospective important pathways.

These were (1) HPG27_526, which encodes a putative uncharacterized AI-2 exporter that was twofold decreased in biofilm cells; (2) HPG27_166, encoding a hypothetical gene that was the most highly upregulated biofilm gene (12-fold upregulated); (3) HPG27_1233, which encodes an adenine methyltransferase that was sixfold upregulated in biofilms; (4) HPG27_1066, which encodes a cytosine methyltransferase that was fourfold upregulated in biofilms; (5) HPG27_715, which encodes a MATE-family uncharacterized efflux pump that was 2.5-fold upregulated in biofilms; (6) HPG27_383/*acsA*, which encodes a putative acetyl-coenzyme A synthase possibly in the same pathway as AcxA, and was twofold upregulated in biofilms and (7) *hypF*, a gene required for producing the Ni-Fe hydrogenase cyanide ligand that was 2.5-fold upregulated in biofilms.

**Fig. 7:**
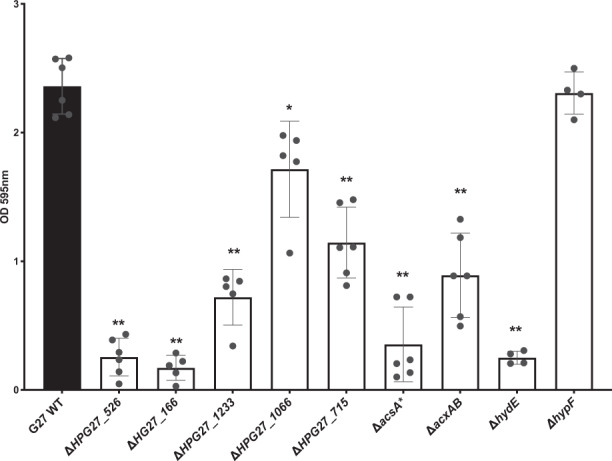
Biofilm formation by *H. pylori* G27 WT and its isogenic mutants. Biofilm formation was assessed using the microtiter plate crystal violet biofilm assay after 3 days of growth. Experiments were performed two independent times with at least six technical replicates for each. Error bars represent standard errors for each average value. Statistical analyses were performed using ANOVA (*P < 0.01; **P < 0.0001).

Each of these genes was replaced with either a chloramphenicol or a kanamycin resistance gene. The loss of these genes did not affect growth except for Δ*acsA*, which demonstrated a slight delay in growth. After constructing and verifying each mutant, biofilm formation was evaluated using the crystal violet assay. 8/9 mutants demonstrated substantially reduced biofilm formation (Fig. 7). The most substantial defects occurred with loss of genes acting in the AI-2, hydrogenase, and acetone metabolism pathways, strongly supporting these processes are critical for biofilm formation. The results with the putative AI-2 transporter mutant (HPG27_526) were surprising because this gene was downregulated in the biofilm transcriptomics, and thus we predicted that loss might result in elevated biofilm. However, this gene product has yet to be characterized in *H. pylori*, so it may play different or additional roles, or AI-2 may play a more nuanced role in biofilm formation. Interestingly, the two hydrogenase-related genes had different effects: the *hydE* mutant had a substantial biofilm defect, while the loss of *hypF* biofilm-upregulated gene had no biofilm formation defect. Overall, however, these results support that transcriptomics and transposon mutant screens are powerful ways to identify genes that are important for *H. pylori* biofilm formation.

### Acetone metabolism is essential to *H. pylori* biofilm formation on AGS surface

**Fig. 8:**
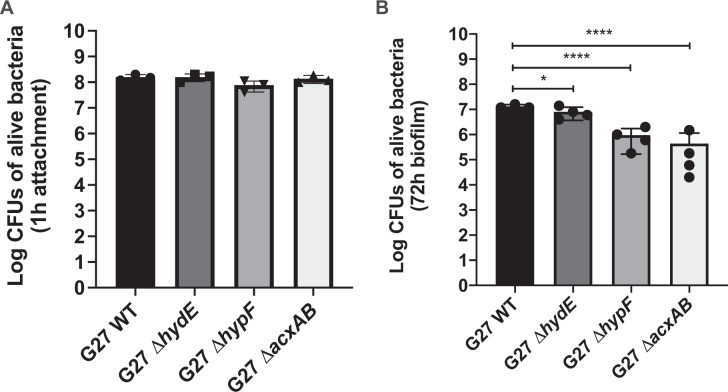
Biofilm formation by *H. pylori* G27 WT and its isogenic mutants on AGS cells surface. Biofilm formation was performed in 24-well plates with 85% AGS cells confluency. Bacterial numbers were determined by plating (CFU/ml) after 1 h (**A**) and three days (**B**) of coincubation. Experiments were performed three independent times with triplicates each time. Error bars represent standard deviations for each average value. Statistical analyses were performed using one-way ANOVA with Tukey post hoc test (*P < 0.05, ****P < 0.0001).

We next asked whether the same genes are required to construct biofilms on biotic surfaces as are required on abiotic surfaces. Since the genes on the pathway of hydrogen and acetone metabolism were identified in both transposon library screening and transcriptomic analysis, we focused on the biofilm-forming ability of three mutants in this pathway (*ΔacxAB*, *ΔhydE*, and *ΔhypF*) on the AGS cells surface. As done with wild type and flagellar mutants above, we first investigated whether these mutants had defects in AGS cell attachment by monitoring bacterial numbers are 1 h of coincubation. All three mutants attached to the AGS cell surface at the same level as that of WT (Fig. 8A), suggesting they do not have a defect in initial attachment in contrast to flagella-deficient mutants (Fig. 4A). After a three-day incubation, the phenotypes varied. The mutant lacking acetone carboxylase, Δ*acxAB*, formed significantly less biofilm compared to wild type (~150-fold less) (Fig. 8B). The two hydrogenase-related mutants, lacking *hydE* or *hypF*, performed distinctly in biofilm formation. Loss of *hydE* had less effect on biofilm formation, while the *hypF* mutant was more severely defective, akin to the *ΔacxAB* mutant (Fig. 8B). Interestingly, the *ΔhydE* and *ΔhypF* mutants performed in an opposite manner on abiotic surface (Fig. 7), suggesting that the encoded enzymes serve different roles when *H. pylori* establishes biofilm on various types of surface. Together, these results suggest that some processes, such as acetone metabolism, are essential for *H. pylori* biofilm formation in general and some processes are biofilm-context dependent.

## Discussion

We report here characterization of biofilm formation in *H. pylori*, using strain G27, which forms robust biofilms under many conditions. We identified genes required for and upregulated during biofilm growth. Several of these genes matched previous reports, but new properties were also shown to be key for biofilm formation, including acetone metabolism, hydrogen utilization, DNA methylation, and multiple genes whose products have unknown functions. Overall, our results suggest several universal aspects of *H. pylori* biofilm growth important on multiple surfaces, as well as properties that appear to vary in importance across models.

*H. pylori* G27 forms robust biofilms that consist of reproducible 3-dimensional and dense packed-cell structure under standard *H. pylori* growth conditions. Numerous studies support that *H. pylori* G27 forms biofilms on abiotic surfaces^14,16,26,29,30,50^, and here we expand those studies to biofilms on epithelial cells, referred to as biotic surfaces. The biofilms formed on biotic surfaces were similar to those on abiotic surfaces in that there were layers of bacteria that were tightly packed and enmeshed in flagellar filaments (Fig. 3). Biotic biofilms differed in that the cells were mostly spiral or rod-shaped (Fig. 3), versus nearly fully coccoid in the abiotic biofilms (Fig. 2). This appearance is consistent with the idea that the abiotic biofilms are under nutrient depletion stress; previous work showed that planktonic cells cultured for this time were also mostly coccoid^16^. Indeed, previous work characterized growth of an *H. pylori* G27 derivative on epithelial cells, finding the bacteria grew while directly attached into microcolonies that consisted of tightly packed spiral-shaped cells^33,34^. These authors elegantly showed that the *H. pylori* obtain nutrients directly from the cells, including iron, a finding that agreed well with previous reports^51^. Latter work also showed *H. pylori* G27 formed cell-associated microcolonies^29^. Initially, these microcolonies were not characterized as biofilms^33,34^, but we propose that they are in line with Anderson et al.^28^. This idea is based on the fact that there are multiple layers of tightly packed bacteria, attached to a surface and each other, with flagella filament as extracellular structures promoting adherence. These results, in summary, support that *H. pylori* G27 is an excellent model for both abiotic and biotic biofilm formation.

One finding from this work is confirmation that flagella are key for multiple types of *H. pylori* biofilms, consistent with previous findings on abiotic surfaces^16,38^ and also studies with *E. coli*^52,53^. Both biotic- and abiotic-surface biofilms contained a meshed network of flagella filaments that promoted biofilm formation, likely playing a structural role to hold bacteria together and to the surface. Mutants lacking flagella showed poor epithelial cell adherence and biotic biofilm formation. In comparison, mutants that had non-functional flagella formed significantly more biofilm compared to isogenic Fla^−^ cells, but did not match wild-type *H. pylori*. This outcome suggests that active motility is required for full adherence and biofilm formation in this model. Consistent with the importance of flagella, biofilm cells over-expressed flagellar genes: here we show *rpoN* and *flaA*, while previous work reported additional flagellar genes^16^. Furthermore, two of eight biofilm-defective transposon mutants affected flagella, *faaA* and *fliK*. FaaA encodes a large 348 kD protein of the autotransporter family predicted to have a beta helix structure that would extend substantially from the cell surface^46^. FaaA localizes to the flagellar sheath is plays a role in flagellar stability^46^. Therefore, the *faaA* mutant may have biofilm defects due to loss of flagella or to other properties of FaaA. FliK is a hook-length control protein, important for normal flagella formation. Mutants lacking *fliK* form structures that consist of long flagellar hooks, so-called polyhooks^45^, and our results suggest these hooks are not adequate to promote biofilm formation. Our studies thus support that flagella continue to be expressed in *H. pylori* biofilms and contribute to their formation on both abiotic and epithelial surfaces.

Our biofilm-defective transposon mutant screen found evidence that both acetone and hydrogen utilization are important for biofilm formation. The *acxA* transposon mutant and *acxAB* defined mutants showed a significant defect on both abiotic and biotic surfaces (Figs 5, 7, and 8), and the *acxABC* operon was significantly upregulated in biofilm cells (Table 2 and Supplementary Table 2). These gene products are predicted to catalyze the ATP-dependent carboxylation of acetone to acetoacetate, which creates two molecules of acetyl-CoA^44^. The *H. pylori* acetone carboxylase shows high-sequence identify (59–68% amino acid identity) to characterized acetone carboxylases, and thus is predicted to share the same function. Several microbes use acetone as a carbon and energy source, although this has not been shown for *H. pylori*. *H. pylori acxABC* contributes to gastric colonization, in mice and there is acetone in the gastric tissue^44^. Additional work will be needed, however, to characterize the acetone source during biofilm growth and confirm the function of these gene products.

Another alternative type of biofilm-related metabolism we found here in multiple ways is the use of hydrogen as an electron donor via *H. pylori*’s Ni-Fe hydrogenase. Hydrogenase is created by the products of the *hydABCDE*, *hypA*, *hypBCD*, and *hypEF* operons. *hydAB* encodes the main hydrogenase subunits, *hydC* encodes the cytochrome b subunit, and *hydD* is required for hydrogenase maturation. *hydE* has an unknown function in *H. pylori*, but it is known that *hydE* mutants lose hydrogenase activity, are not rescued by exogenous nickel, and have normal urease activity^48^. Thus, *hydE* mutants lack hydrogenase function, and because *hydE* is the last gene in the operon, mutations should not have polar effects. The five *hyp* genes (*hypA-F*) are required for cofactor maturation in support of hydrogenase and/or urease. HypE and HypF are required only for hydrogenase activity^48,54^, and in heterologous systems, produce the non-protein CN and CO ligands that hold the hydrogenase Fe^55^. The *hypE* mutant hydrogenase phenotype is corrected by exogenous nickel, but it is not known if the *hypF* mutant shares this property^48^. These results thus suggest that *hypF* mutants may have conditional loss of hydrogenase activity, recovering under high Ni. Our results found that *hydE* mutants were strongly defective in abiotic biofilms, while *hypF* mutants were more defective in biotic biofilms. It’s difficult to speculate why these differences exist in part because neither of these encoded proteins are well characterized in *H. pylori*. The results do, however, suggest that hydrogenase activity plays a role in biofilm formation and that some different properties may be needed between these two types of biofilms.

Biofilm *H. pylori* G27 displayed a distinct transcriptome, similar to that reported for strain SS1^16^. Several types of genes were similarly regulated, such as downregulation of TCA cycle genes and upregulation of flagellar genes. There was, however, minimal overlap in the exact genes that were differentially expressed in the biofilms of the two strains. This discrepancy may be due in part to the experimental design differences, in that the SS1 study employed planktonic and biofilm cells in the same culture wells, while the work here used different culture conditions because of the soft G27 pellicle that could not be easily separated during sample preparation. One gene, *lptB*, was upregulated ∼3-fold in both SS1 and G27 biofilms. *lptB* encodes a lipopolysaccharide export system ATPase, agreeing well with the observation that LPS components are part of *H. pylori* biofilm matrix^11,30^. Two additional LPS-related genes were upregulated in these studies: G27 *rfaJ-1*, which encodes an α-1,6-glucosyltranferase that plays an integral role in the biosynthesis of the core LPS^56^, and SS1 *lpxB*, which encodes a lipid-A-disaccharide synthase^16^. Previous work similarly highlighted the importance of LPS, finding that loss of some LPS-related genes, lpxF and lpxL, promoted biofilm formation^30^. The expression of the gene encoding for LpxF was downregulated in this study. Overall, these results show that LPS regulation is a key aspect of *H. pylori* biofilms.

*H. pylori* G27 biofilm cells displayed pronounced metabolic changes, including downregulation of glycolysis, the TCA cycle, and specific electron transport chain components. There were seven downregulated genes spread throughout the central metabolic pathways. These included the glycolytic enzyme glucokinase (*glk*); the TCA enzymes citrate synthase (*gltA*), isocitrate dehydrogenase (*icd*), and fumarase (*fumC*); the TCA-related enzyme alpha-ketoglutarate oxidoreductase (*oorD*), which catalyzes the conversion of α-ketoglutarate to succinyl coenzyme A; and the electron transport chain components NADH-ubiquinone oxidoreductase (*nqo10*) and NADH oxidoreductase I (*nuoB*) (Table 3 and Supplementary Table 3). Genes related to energy metabolism, including ferredoxin (HPG27_256), thioredoxin (*trxA*), and the super dismutase *sodB* were among the most downregulated genes in our biofilm cells. Interestingly, these same genes were also downregulated in other studies, after adhesion to gastric cells in vitro^42^. Altogether, these data suggest that cells grown in biofilm may decrease flux through glycolysis and the TCA cycle as a way to lower or alter metabolism. This lowered growth state may also contribute to antibiotic tolerance, as described for other organisms^7–10^.

In addition to the pathways described above, the transcriptomics analysis highlighted several other interesting gene expression patterns of *H. pylori* G27 biofilm cells. The first was upregulation of the *cag* type IV secretion system (*cag*-T4SS) genes. *H. pylori* G27 biofilms expressed high amounts of the genes for the CagB ATPase, and Cag4, encoding a cell wall hydrolase of the *cag* T4SS. Previous work showed *H. pylori* strain SS1 biofilms expressed high amounts of the genes for the CagE ATPase, the CagL pilus protein, and the CagW protein of unknown function^16^. Consistent with these findings, biofilm proteome analysis identified upregulation of the CagA and CagD *cag*-T4SS proteins during biofilm formation^23^, or found that *cagE* is required for biofilm production^57^. While there is little overlap between the genes identified in these studies, the repeated overexpression or requirement for *cag*-T4SS genes in *H. pylori* biofilms suggest that the *cag*-T4SS plays an important role in this growth state. This idea is intriguing, as to date, the only known function of the *cag*-T4SS is in proinflammatory macromolecule delivery.

Our data also showed that several genes of one of the other *H. pylori* T4SS’s were also upregulated in biofilm cells. This T4SS system, along with T4SS and ComB system, has been suggested to play a role in DNA uptake^58^. We found that many of the *tfs4* system genes were upregulated, including *virC1*, *virD4, virb11-like*, HPG27_977, HPG27_972, HPG27_971, HPG27_969, and HPG27_968 (Supplementary Fig. 5). This extensive upregulation suggests these genes might be co-regulated by an as-yet-unknown regulator. The role of *tfs4* during biofilm formation is not yet understood but it could increase DNA transfer between biofilm cells. This idea is supported by recent data from *Bacillus subtilis*, where biofilm formation drove high rates of conjugative ICE transfer compared to planktonic cells^59^. Such a situation might also increase the requirement for restriction-modification (R-M) systems, of which several were upregulated (Table 2 and Supplementary Table 2) or required for biofilm formation (Fig. 7).

Taken together, our genetic and transcriptomic analyses suggest that *H. pylori* adjusts its phenotype when grown as biofilm, changing its metabolism to one that decreases central metabolism and benefits from alternate sources. *H. pylori* G27 can form a developed biofilm containing a mesh of flagella on gastric epithelial cells that resembles the one formed on abiotic surfaces. Flagella played a key structural role in both situations. In addition, we used genetic screens and transcriptomics to find new genes associated with *H. pylori* biofilm including genes encoding for acetone metabolism, hydrogen utilization, the *cag* T4SS and the relatively uncharacterized T4SS-4. Overall, it’s clear that the *H. pylori* biofilm is a growth state that *H. pylori* adopts under multiple conditions, requires a myriad set of abilities to create, and likely results in *H. pylori* with distinct physiology, including resistance to multiple antibiotics^20–22^.

## Methods

### Bacterial strain and growth conditions

This study employed *H. pylori* wild-type and clinical strains, mutants generated using a transposon library that was constructed in strain G27 and generously provided by Nina Salama^60^, as well as mutants generated for this work (Table 4). Strains were grown on Columbia Horse Blood Agar (CHBA) (Difco), containing: 0.2% β-cyclodextrin, 10 μg of vancomycin per ml, 5 μg of cefsulodin per ml, 2.5 U of polymyxin B per ml, 5 μg of trimethoprim per ml, and 8 μg of amphotericin B per ml (all chemicals are from Thermo Fisher or Gold Biotech), or Brucella broth (Difco) containing 10% heat-inactivated fetal bovine serum (BB10; Gibco/BRL). Cultures were grown under microaerobic conditions (10% CO_2_, 5% O_2_, 85% N_2_) at 37 °C. For antibiotic resistance marker selection, bacterial media were supplemented with 25 μg of chloramphenicol (Cm) per ml or 75 μg kanamycin (Km) per ml. For growth curves, overnight cultures were diluted to OD 0.2 in fresh media and growth measurement were performed using an automatic microplate reader (Tecan Infinite F200), shaking at 3 g, until 24 h. OD600 readings were taken every 15 min with continuous shaking between readings.

**Table 4:** Strains used for this in the present study.

*Strain*	KO reference no.	Genotype or description	Reference and/or source(s)
*G27*	KO379	WT strain	N. Salama (Fred Hutchison Cancer Research Center, USA)
*SS1*	KO457	WT strain	J. O’Rourke (University of New South Wales, Australia)
*J99*	KO479	WT strain	N. Salama (Fred Hutchison Cancer Research Center, USA)
*26695*	KO668	WT strain	ATCC 700392
*X472AL*	KO612	WT strain	D. Berg (Washington University, USA)
*M6*	KO613	WT strain	E. Joyce, A. Wright (Tufts University School of Medicine, USA)
*CYP34 (CYP3401)*	KO637	WT strain	D. Berg
*HP43504*	KO669	WT/clinical isolate	NTCT 11637
*HP 100-1*	KO763	WT/clinical isolate	J. Solnick (UC Davis, USA)
*HP 12-1*	KO760	WT/clinical isolate	J. Solnick
*HP 28-1*	KO761	WT/clinical isolate	J. Solnick
*HP116-1*	KO764	WT/clinical isolate	J. Solnick
*HP 125-2*	KO765	WT/clinical isolate	J. Solnick
*HP 208-2*	KO767	WT/clinical isolate	J. Solnick
G27 Δ*HPG27_166*^***a***^	KO1635	Δ*HPG27_166:aphA3*	This study
G27 Δ*HPG27_526*^***a***^	KO1637	Δ*HPG27_526::cat*	This study
G27 Δ*hydE*^***a***^	KO1638	Δ*hydE::cat*	This study
G27 Δ*HPG27_1233*^***a***^	KO1639	Δ*HPG27_1233::cat*	This study
G27 Δ*HPG27_1066*^***a***^	KO1640	Δ*HPG27_1066::cat*	This study
G27 Δ*acsA*^***a***^^*,*^^***b***^	KO1641	Δ*acsA::cat*	This study
G27 Δ*acxAB*^***a***^	KO1642	Δ*acxAB::cat*	This study
G27 Δ*HPG27_715*^***a***^	KO1644	Δ*HPG27_715::cat*	This study
G27 *ΔmotB*	KO489	*ΔmotB*::kan-sac2	This study
G27 *ΔfliA*	KO1285	*ΔfliA*::kan	K. Guillemin (University of Oregon, USA)

*WT* wild-type, *aphA3* kanamycin resistant, *cat* chloramphenicol resistant, *ATCC* American Type Culture Collection.

^a^All transformations were performed using natural transformation using plasmid constructs (pTwist_Amp) in which the gene’s coding sequence was completely or partially removed and replaced by a kanamycin or chloramphenicol-resistance cassette using allelic exchange.

^b^Mutant Δ*acsA* showed a slight defect in growth.

### Biofilm formation on abiotic surfaces and crystal violet assay

*H. pylori* strains were grown overnight with shaking in BB10, diluted to an OD600 of 0.15 with fresh BB10 media and 200 μl of the culture was used to fill triplicate wells of a sterile 96-well polystyrene microtiter plate (Costar, 3596). Wells at the edges of the microplate were filled with 200 μl of sterile water to avoid evaporation during incubation.

Following static incubation of three days under microaerobic conditions, culture medium was removed by aspiration and the plate was washed twice using 1x phosphate-buffered saline (PBS). The wells were then filled with 200 μl of crystal violet (0.1%, wt/vol), and the plate was incubated for 2 min at room temperature. After removal of the crystal violet solution by aspiration, the plate was washed twice with PBS and air dried for 20 min at room temperature. To visualize biofilms, 200 μl of 70% ethanol (vol/vol) was added to the wells and the absorbance at 595 nm was measured.

### Confocal laser-scanning microscopy

Biofilms of *H. pylori* G27 and mutants were prepared as described above using BB10 media, however, for confocal laser-scanning microscopy (CLSM), μ-Slide 8-well glass bottom chamber slides (ibidi, Germany) were used instead of 96-well microtiter plates. Three-day-old biofilms were stained with FM^®^1–43 (Invitrogen) or FilmTracer LIVE/DEAD biofilm viability kit (Invitrogen) according to the manufacturer’s instructions. Stained biofilms were visualized by CLSM with an LSM 5 Pascal laser-scanning microscope (Zeiss) and images were acquired using Imaris software (Bitplane). Biomass analysis of biofilm was carried out using FM^®^1–43 stained z-stack images (0.15 μm thickness) obtained by CLSM from randomly selected areas. The biomass of biofilms was determined using COMSTAT (27).

### Cell culture

AGS (ATCC CRL 1739) human gastric epithelial cells were obtained directly from the American Type Culture Collection (ATCC) and maintained in RPMI-1640 medium (RPMI, Gibco) containing 10% heat-inactivated fetal bovine serum (FBS, Gibco) at 37 °C under 10% CO_2_. For co-culture with *H. pylori*, AGS cells were maintained in DMEM/Ham’s F-12 medium (Gibco) with 10% FBS.

### AGS cell attachment and biofilm formation

For assessment of AGS cell attachment and biofilm formation, *H. pylori* were maintained on CHBA, scrapped from the plate, resuspended in BB10 medium, and grown overnight with shaking. After an overnight growth, bacterial cells were harvested by centrifugation and resuspended in a pre-warmed mixture of DMEM/F-12 Medium with 10% FBS at a concentration of 1 × 10^8^ CFU/ml. AGS cells were seeded in 24-well culture plates and cultured to reach 85–90% confluency. Number of cells were estimated using a hemocytometer. The AGS cell RPMI-1640 media was replaced by 1 ml fresh resuspended *H. pylori* culture at a multiplicity of infection (MOI) of 10. Attachment was assessed after a total of 1 h of coincubation. *H. pylori* was added without centrifugation and incubated at 37 °C under 10% CO_2_ for 1 h. However, since flagella and motility might play a role in surface colonization, we also tested *H. pylori* upon a 250 × *g* centrifugation for 30 min to force bacterial cells being in contact with AGS cells and then incubated at 37 °C under 10% CO_2_ for another 30 min. For biofilm formation, *H. pylori* was added without centrifugation and incubated at 37 °C under 10% CO_2_ for 3 days. The *H. pylori*-AGS cell culture was daily washed with 1 x PBS (twice) to remove unattached *H. pylori* cells. *H. pylori*-AGS cell culture was collected by trypsinization (trypsin/EDTA (Gibco)) and harvested in fresh DMEM/Ham’s F-12/FBS media. Bacterial sample was plated to determine the bacterial numbers.

### Scanning electron microscopy

*H. pylori* wild-type G27 was grown on round glass coverslips in 6-well plates (12 mm, Costar) by dispersing 4 ml of a culture diluted to OD 0.15 in BB10. For biofilm formed on biotic surface, AGS cells were first maintained in DMEM/Ham’s F-12 medium with 10% FBS in 6-well plates containing glass coverslips. Once confluent, cells were washed twice with 1 x PBS and 1 × 10^8^ CFU/ml of *H. pylori* wild-type G27 culture in mixture DMEM/F-12 Medium with 10% FBS was added to the wells.

For both cultures, plates were incubated for 3-days under microaerobic conditions. Glass coverslips containing biofilm alone or AGS cells and *H. pylori* were washed twice with PBS and fixed with 2.5% glutaraldehyde (v/v) for 1 h at room temperature. Samples were then dehydrated with ethanol (10%, 25%, 50%, 75%, 90%, 100%), critical point dried (model: blazers Union 342/11 120B), sputtered with ~20 nm of gold (model: Technics Hummer VI) and imaged with a FEI Quanta 3D Dual beam SEM operating at 5 kV and 6.7 pA at the SEM facility at University of California Santa Cruz.

### Screening approach for biofilm-defective mutants

A *Tn*-7-based *H. pylori G27* transposon mutant library^43^ was grown overnight in BB10 supplemented with 25 μg of chloramphenicol, diluted to an OD600 of 0.15 and then used to fill duplicate wells of a 6-well plate (Costar® 3516, Corning, Corning, NY, USA). After an hour of incubation, media containing non-attached, planktonic bacteria and/or potential biofilm-defective mutants was removed, and transferred to a new sterile 6-well plate and incubated again. The procedure was repeated at different incubation time (i.e., 2, 3, 24, 48, or 72 h). After 72 h, this enriched biofilm-defective sample was plated on CHBA media and individual colonies were isolated and stored at −80 °C. A total of 97 potential biofilm-defective mutants was isolated from this enrichment approach, and subsequently tested for abnormal biofilm formation using the crystal violet biofilm assay described above.

### Identification of transposon interrupted genes

Nested PCR was used to identify the site of transposon insertions in biofilm-defective mutants, using methods described in Salama et al.^43^. For the first round of PCR, random primers with constant 5′ tail regions in tandem with a transposon-specific primer (Upstream or Downstream) were used (Supplementary Table 4). For the second round, a primer specific to the transposon was utilized with a primer complementary to the tail region of the original random primer. Therefore, the final products contain a portion of the transposon along with surrounding genomic information. These PCR products were sequenced (Sequetech, Mountain View, CA) and then compared to the G27 genomic sequence^49^ at the UCSC Microbial Genome Browser (www. http://microbes.ucsc.edu).

### Biofilm and planktonic growth conditions for transcriptomic analysis

*H. pylori* G27 cells for transcriptomics were collected either from biofilm or from planktonic cultures under microaerobic conditions. In each case, cells were harvested by centrifugation (10 min, 1500 × *g*), washed two-times with ice cold PBS, and resuspended in 1 ml of Trizol Max (Ambion Bacterial Enhancement Kit, Ambion, Life technology, Carlsbad, CA, USA). For biofilm cells, *H. pylori* (4 ml per well) was grown in BB10 in 6-well plates (Costar), without shaking, for three days, and then scrapped from the surface with a sterile cell scraper. For planktonic cells, *H. pylori* was grown in BB10 in glass flasks with shaking for 24 h. Please note that the majority of both the planktonic (24 h) and biofilm (72 h) grown cultures are in the coccoid form.

### RNA extraction and library construction

Total RNA from *H. pylori* in Trizol Max was extracted using the Trizol Max Bacterial Enhancement Kit (Ambion, Life Technology, Carlsbad, CA, USA) as described by the manufacturer. RNA was further purified and concentrated using an RNAeasy Kit (Qiagen). rRNA was removed using RiboZero magnetic kit (Illumina). Sequencing libraries were generated using NEBNext Ultra^TM^ Directional RNA library Prep Kit for Illumina (NEB, USA). Complementary DNA (cDNA) library quality and amount were verified using Agilent Bioanalyzer 2100 system (Agilent technologies, CA, USA) and then sequenced using Illumina NextSeq Mid-Output (UC Davis Genome Center).

### Transcriptomic analysis

RNA-seq data were analyzed using CLC Genomics Workbench (version 11.0, CLC Bio, Boston, MA, USA). All sequences were trimmed, and forward and reverse sequenced reads generated for each growth state (biofilm vs. planktonic; three biological replicates for each condition) were mapped against the G27 reference genome^49^ to quantify gene expression levels for each experimental condition. The expression value was measured in Reads per Kilobase Per Million Mapped Reads (RPKM). Genes were considered as differentially expressed when log2 (fold-change) was above 1 or below −1 and with statistical significance (*P*-value < 0.05, false-discovery rate (FDR < 0.001).

### Quantitative PCR

To validate RNA-seq data, qRT-PCR was performed to quantify the transcription of six selected genes (we randomly selected two upregulated; *virC1* and *virB11*, two downregulated; *fdxB* and HPG27_526 and two non-differentially expressed genes; *lctP* and *hopC*). Total RNA from three independent experiments was obtained and used for qRT-PCR. Primers were designed using Primer-Blast (https://www.ncbi.nlm.nih.gov/tools/primer-blast/) and are listed in Supplementary Table 4. The same amount of total RNA (1 μg/μl) was reverse transcribed using the LunaScript^TM^ RT SuperMix Kit (NEB) and qPCR reactions were prepared using Luna Universal qPCR Master Mix kit (NEB) The run was performed in Connect^TM^ Thermal Cycler (Bio-Rad) with the following cycling parameters: 2 min at 95 °C, followed by 39 cycle of 30 s at 95 °C, 30 s at 60 °C, and 30 s at 72 °C. Relative expression of each gene was normalized to that of the 16S gene, whose expression was consistent throughout the different conditions. Quantitative measures were made using the 2^−ΔΔCT^ method^61^. Three biological replicates and two technical replicates of each condition were performed.

### Design and construction of mutants

Clean gene knockout constructs were designed to fully replace the target gene’s coding sequence with a chloramphenicol-resistance cassette (*cat*) or a kanamycin resistance cassette (*aphA3*), and synthesized by Twist Bioscience (San Francisco, CA). All constructs (Table 1 and Supplementary Table 1) were cloned in the pTwist Amp high copy vector and transformed into *Escherichia coli* DH10B. Purified vector containing the construct (2 to 10 μg) was used to transform *H. pylori* G27 WT. Mutants were selected on chloramphenicol or kanamycin-containing plates, colony purified, and confirmed by PCR using primers that flank antibiotic cassette (Supplementary Table 4).

### Statistical analysis

Biofilm data were analyzed statistically using GraphPad Prism software (version 7, GraphPad Software Inc., San Diego, CA) by application of Wilcoxon–Mann–Whitney test or one-way ANOVA with Bartlett’s test. *P* < 0.05 or <0.01 were considered reflecting statistically significant.

### Reporting summary

Further information on research design is available in the Nature Research Reporting Summary linked to this article.

## Supplementary information

Supplementary Information

Reporting Summary

## Data Availability

The data sets generated during and/or analyzed during the current study are either shown in the manuscript or available from the corresponding author on reasonable request.
