# Supplementary material for: Genetic requirements and transcriptomics of Helicobacter pylori biofilm formation on abiotic and biotic surfaces
Source: NPJ Biofilms Microbiomes. 2020 Nov 27;6:56. doi: 10.1038/s41522-020-00167-3 (PMC7695850; doi:10.1038/s41522-020-00167-3)
Supplement: Supplementary file 1 — Supplementary Information [file 41522_2020_167_MOESM1_ESM.pdf]

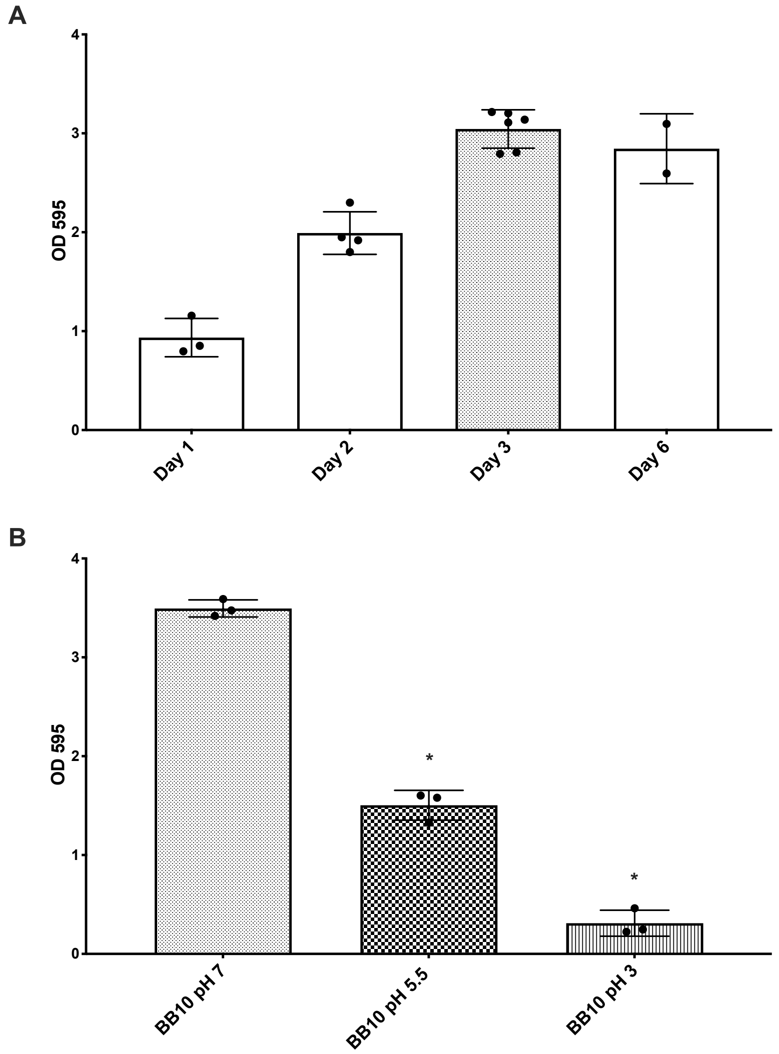

Supplementary Figure 1. ***H. pylori* G27 biofilms is time and pH-dependent.** (A) Biofilm formation of *H. pylori* strains were assessed using the microtiter plate biofilm assay at different time points. (B) Effect of pH variation on biofilm development, using BB10 pHed to different values and its normal, pH 7. Results represent the crystal violet absorbance at 595nm which reflect the biofilm biomass. Experiments were performed three independent times with at least 3 technical replicates for each. Error bars represent standard errors of the mean for each average value. Statistical analyses were performed using ANOVA (*, P <0.05)

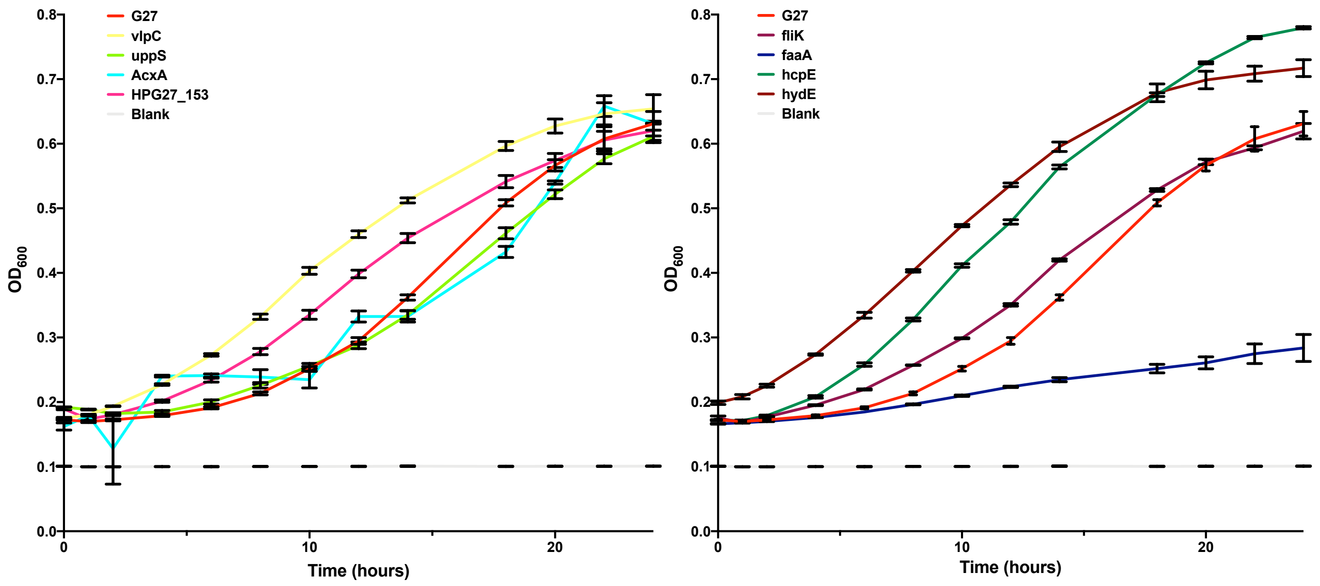

Supplementary Figure 2. Growth curves of WT G27 strain and biofilm-defective mutants. Overnight cultures were diluted to OD 0.2 into fresh media under agitation (Tecan Infinite F200, 360rpm). Optical densities were monitored by measured at A 600nm at various time points. The data shown are mean ± SD of triplicate experiment.

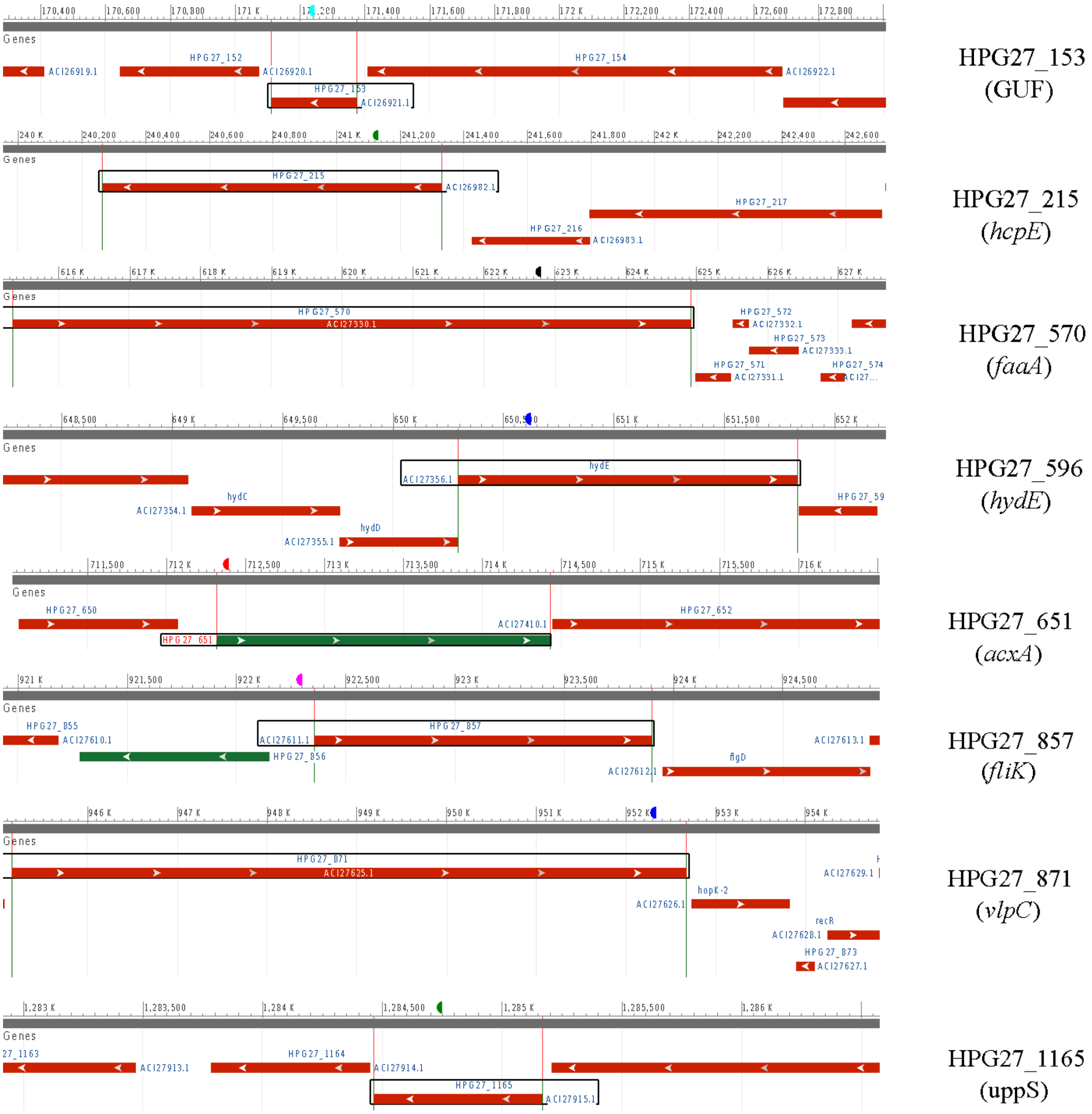

Supplementary Figure 3. Graphical map showing the position and surrounding regions of the insertion site of Tn7 transposon in biofilm-defective mutants. Boxes represent the flanking open reading frame and colored line represent the genomic position of the transposon insertion.

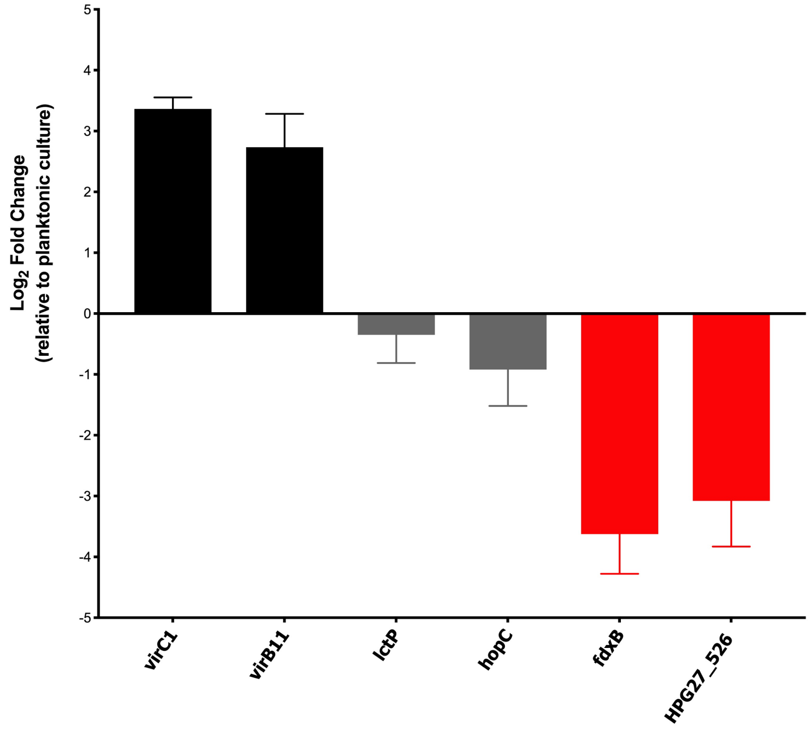

Supplementary Figure 4. qPCR validation of the transcription of selected differentially expressed genes. The data indicate the fold change in expression of genes in H. pylori biofilm cells compared to planktonic cells. Fold changes in gene expressions were calculated after normalization of each gene with the constitutively expressed gene control *gapB*. Bars represent the mean and error bars the standard error of the mean. Black and gray bars represent qPCR and RNA-seq results, respectively. Statistical analyses were performed using threshold cycle ($2^{-\Delta\Delta CT}$) values, and all results were statistically significant (P <0.01) except for *lctP* and *hopC* gene transcripts.

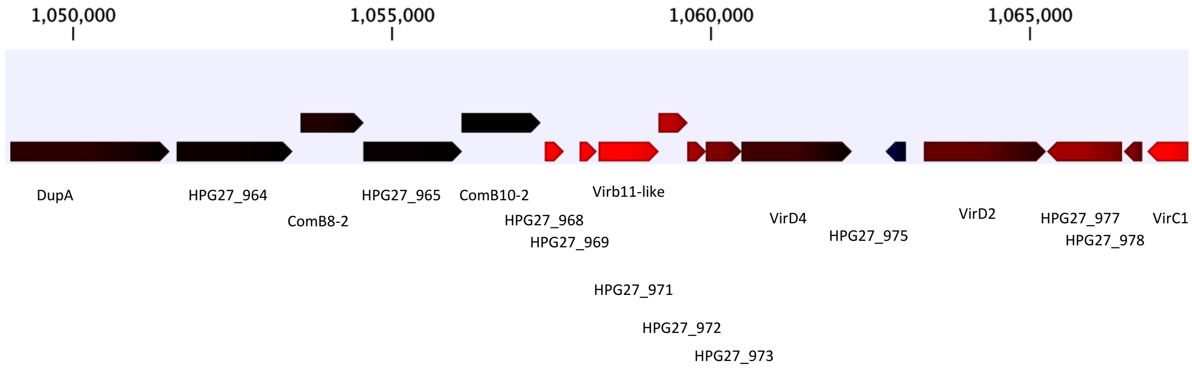

Figure S5. Genes expression of Type IV secretion system 4 (tfs4) genes in *H. pylori* G27 cell grown in biofilm condition. Red represent genes that are up-regulated in cells grown in biofilm condition and higher intensity of red denotes greater fold-change.

Supplementary Table 1.

List of plasmids used in the present study.

Plasmid	**Description**	**Source**
pTwist_Amp 526	Clonig pTwist vector harboring_*HPG27_526::Cm^R*, Amp^R	This study
pTwist_Amp _166	Clonig pTwist vector harboring_*HPG27_166::kan^R* , Amp^R	This study
pTwist_Amp _715	Clonig pTwist vector harboring_*HPG27_715::Cm^R* , Amp^R	This study
pTwist_Amp _1233	Clonig pTwist vector harboring_*HPG27_1233::Cm^R*, Amp^R	This study
pTwist_Amp _1066	Clonig pTwist vector harboring_*HPG27_1066::Cm^R* , Amp^R	This study
pTwist_Amp_ΔacsA	Clonig pTwist vector harboring_*acsA::Cm^R* , Amp^R	This study
pTwist_Amp_ΔAcxAB	Clonig pTwist vector harboring *acxAB::Cm^R* , Amp^R	This study
pTwist_Amp_ΔhydE	Clonig pTwist vector harboring *hydE::Cm^R* , Amp^R	This study

Supplementary Table 2.

Full list of up-regulated genes in *H. pylori* G27 grown in biofilm condition (cutoff ratio ≥ 1 log2 fold change and p-value <0.05) using RNA-seq analysis. These genes are all expressed to higher levels in the biofilm cells.

Name	Putative gene product	Fold change
HPG27_166	Hypothetical protein	12.13
HPG27_979	VirC1	9.07
HPG27_970	VirB11-like	6.43
HPG27_1233	^{m6}A methyltransferase	6.35
HPG27_969	Hypothetical protein	5.93
HPG27_1522	Hypothetical protein	4.72
HPG27_270	Chorismate mutase	4.59
HPG27_968	Hypothetical protein	4.46
HPG27_509	Glutamate racemase	3.92
HPG27_1066	^{m5}C methyltransferase	3.89
HPG27_324	RluA, pseudouridine synthase D	3.57
HPG27_1059	Hypothetical protein	3.53
HPG27_335	Hypothetical protein	3.39
HPG27_269	LysA, diaminopimelate decarboxylase	3.29
HPG27_671	LptB, ABC transporter	3.26
HPG27_506	CagB, cag island protein B	3.18
HPG27_271	Hypothetical protein	3.17
HPG27_442	^{m5}C methyltransferase	3.15
HPG27_834	Hypothetical protein	3.13
HPG27_669	Hypothetical protein	3.06
HPG27_1331	Hypothetical protein	2.97
HPG27_377	TsaB, tRNA A37 threonylcarbamoyladenosine modification protein	2.96
HPG27_72	PrfA, peptide chain release factor RF-1	2.96
HPG27_1241	Thiamine pyrophosphokinase	2.73
HPG27_652	AcxB, acetone carboxylase subunit alpha	2.69
HPG27_589	Hypothetical protein	2.68
HPG27_800	Hypothetical protein	2.63
HPG27_571	ABC transporter, permease	2.62
HPG27_47	HypAV, type II restriction enzyme	2.59
HPG27_1299	HpyAIV, type II restriction endonuclease	2.59
HPG27_660	Hypothetical protein	2.57
HPG27_272	PabB, para-amino benzoate synthase	2.54
HPG27_670	RpoN, RNA polymerase sigma-54 factor	2.53
HPG27_1524	Hypothetical protein	2.53
HPG27_1370	Hypothetical protein	2.53
HPG27_441	^{m5}C methyltransferase	2.53
HPG27_1526	Hypothetical protein	2.53

HPG27_715	Multidrug and toxic compound extrusion protein (MATE)	2.52
HPG27_538	Hypothetical protein	2.51
HPG27_323	Hypothetical protein	2.51
HPG27_446	HofD, outer membrane protein	2.48
HPG27_971	Hypothetical protein	2.44
HPG27_42	HypF, [NiFe]-hydrogenase maturation protein	2.43
HPG27_1501	Putative outer membrane lipoprotein	2.43
HPG27_1175	DNA polymerase III delta prime subunit	2.41
HPG27_1424	Hypothetical protein	2.41
HPG27_562	FlaA, flagellin A	2.38
HPG27_508	Hypothetical protein	2.38
HPG27_633	Hypothetical protein	2.37
HPG27_915	Hypothetical protein	2.36
HPG27_664	Hypothetical protein	2.36
HPG27_67	UreB, urease beta subunit	2.34
HPG27_310	Hypothetical protein	2.33
HPG27_340	FtsK, septum formation protein/cell division	2.32
HPG27_1381	TrxA, thioredoxin	2.32
HPG27_1353	Hypothetical protein	2.30
HPG27_728	Hypothetical protein	2.25
HPG27_1459	FecA, iron(III) dicitrate transport protein	2.24
HPG27_972	Hypothetical protein	2.23
HPG27_400	Hypothetical protein	2.22
HPG27_66	UreI, urea transporter	2.21
HPG27_960	Hypothetical protein	2.21
HPG27_1240	PnuC	2.18
HPG27_290	HpyHI, type II restriction endonuclease	2.18
HPG27_146	RfaJ-1, α(1,2/3)-glucosyltransferase	2.16
HPG27_383	AcsA, Acetyl-coenzyme A synthetase	2.15
HPG27_977	Hypothetical protein	2.14
HPG27_482	Cag4	2.11
HPG27_252	Hypothetical protein	2.11
HPG27_1162	Hypothetical protein	2.10
HPG27_344	TlyA, hemolysin	2.10
HPG27_157	MoeA, molybdopterin molybdenumtransferase	2.09
HPG27_343	RibC	2.09
HPG27_1280	DnaJ, cochaperone	2.07
HPG27_1373	TdhF, thiophene and furan oxidize	2.07
HPG27_1338	Hypothetical protein	2.06
HPG27_651	AcxA, alpha subunit of acetone carboxylase	2.06
HPG27_462	^{m6}A methyltransferase	2.06
HPG27_1503	Hypothetical protein	2.06
HPG27_1203	Hypothetical protein	2.05
HPG27_946	Hypothetical protein	2.04
HPG27_1418	Hypothetical protein	2.04

HPG27_200	Hypothetical protein	2.04
HPG27_1089	Hypothetical protein	2.04
HPG27_1282	Hypothetical protein	2.04
HPG27_563	Hypothetical protein	2.03
HPG27_64	UreF, urease accessory protein	2.03
HPG27_448	Hypothetical protein	2.02
HPG27_1333	RdxA, oxygen-insensitive NADPH nitroreductase	2.01
HPG27_725	Hypothetical protein	2.01
HPG27_447	Hypothetical protein	2.01
HPG27_1073	Hypothetical protein	2.01
HPG27_546	Hypothetical protein	2.00

Supplementary Table 3.

Full list of the down-regulated genes in *H. pylori* G27 biofilm conditions compared to cells in planktonic conditions (cutoff ratio ≥ 1 log2 fold change and p-value <0.05) using RNA-seq analysis, grouped by functional role categories.

Name	Product	Fold change
HPG27_256	Ferredoxin	-4.39
HPG27_516	Hypothetical protein	-3.01
HPG27_1015	Zinc-metalloprotease	-2.86
HPG27_1271	Hypothetical protein	-2.84
HPG27_1035	Pseudo uridylate synthase I	-2.71
HPG27_1180	Hypothetical protein	-2.62
HPG27_1008	SodB, iron-dependent superoxide dismutase	-2.61
HPG27_591	Mda66, modulator of drug activity	-2.60
HPG27_527	Hypothetical protein	-2.57
HPG27_937	Hypothetical protein	-2.56
HPG27_338	KgtP, alpha-ketoglutarate permease	-2.47
HPG27_1314	Type III restriction enzyme M protein	-2.45
HPG27_277	AibA, AI-2 periplasmic binding protein	-2.41
HPG27_1376	Hypothetical protein	-2.39
HPG27_1044	Glk, glucokinase	-2.36
HPG27_548	OorD, 2-oxoglutarate oxidoreductase subunit	-2.36
HPG27_1205	NADH-ubiquinone oxidoreductase chain A	-2.35
HPG27_783	TrxA, thioredoxin	-2.33
HPG27_1125	Hypothetical protein	-2.32
HPG27_957	Cyclopropane fatty acid synthase	-2.31
HPG27_1329	Biotin synthetase	-2.31
HPG27_887	Hypothetical protein	-2.31
HPG27_521	Ribosomal protein S21	-2.29
HPG27_1156	Hypothetical protein	-2.25
HPG27_1206	NuoB, NADH oxidoreductase I	-2.25
HPG27_1273	FumC, fumarase	-2.22
HPG27_469	Urease-enhancing factor	-2.21
HPG27_549	OorA, 2-oxoglutarate oxidoreductase subunit	-2.19
HPG27_279	DppC, dipeptide transport system permease protein	-2.14
HPG27_1007	Adhesin-thiol peroxidase	-2.14
HPG27_306	FlgH, flagellar L-ring protein precursor	-2.14
HPG27_1269	RpsJ, 30S ribosomal protein S10	-2.13
HPG27_794	DNA-binding protein HU	-2.12
HPG27_1042	Zwf, glucose-6-phosphate dehydrogenase	-2.11
HPG27_629	Hypothetical protein	-2.10
HPG27_1514	YaeE, amino acid ABC transporter	-2.09
HPG27_1517	LpxF, Lipid A 4'-phosphatase	-2.08
HPG27_91	TlpA, methyl-accepting chemotaxis transmembrane sensory protein	-2.08

HPG27_526	Membrane protein belonging to AI-2E family transporter	-2.04
HPG27_1272	Ribonuclease HII	-2.03
HPG27_762	Metalloprotease	-2.03
HPG27_24	Icd, Isocitrate dehydrogenase	-2.02
HPG27_679	L-asparaginase II	-2.00

Supplementary Table 4.

List of primer used in the present study.

Primer	Sequence 5′ to 3′	Product length
Fw_*lctP*	CGCTTGGATTGTGATCGCTG	164
Rv_*lctP*	CGACTAAAATCGCCGCTGTG	
Fw_ HPG27_526	GGGGTCTTGCCTCTTGGAAT	187
Rv_ HPG27_526	CCAAAGACGCTAGGCCGTAT	
Fw_*virC1*	GTTTGCCCAATTTCACGCTCT	181
Rv_*virC1*	ATGTCTAGTTGGCTTGGCGT	
Fw_*virB11*	GGTGGCACAGCTAGTGGTAA	200
Rv_*virB11*	GGAGACATTCTCATAGCCGCA	
Fw_*hopC*	GCGACTGGTTCAGATGGTCA	153
Rv_*hopC*	ATTCCCAATGTTGGCGGCTA	
Fw_16S	ATGGATGCTAGTTGTTGGAGGGCT	185
Rv_16S	TTAAACCACATGCTCCACCGCTTG	
Fw_Cat*	TATGATATAGTGGATAGATTTATGATATAAT	970
Rv_Cat*	TCCGCAGGACGCACTACTCTCGGCAGA	
Fw_Kan*	GGCCGGATCCGATAAACCCAGCGAACC	1410
Rv_Kan*	GGCCAAGCTTTTTAGACATCTAAATC	
CAT Tn7 Up#,§	ACTTTATTGTCATAGTTTAGATCTATTTTG	
CAT Tn7 Dw#,§	ATAATCCTTAAAAACTCCATTTCCACCCCT	
CAT Tn7 Up2*,§	TCAGTTTAAGACTTTATTGTC	
CAT Tn7 Dw2*,§	CAGTTCCCAACTATTTTGTCC	
Random 1#	GGCCACGCGTCGACTAGTACNNNNNNNNNNAGAG	
Random 2#	GGCCACGCGTCGACTAGTACNNNNNNNNNNACGCC	
Random PCR 2$	GGCCACGCGTCGACTAGTAC	

* Primers for antibiotic resistance cassette

First PCR using CAT Tn7 Up or CAT Tn7 Dw with Random 1 or Random 2

$ Second PCR using CAT Tn7 Up2 or CAT Tn7 Dw2 and Random PCR2

§ Salama, Shepherd et al. 2004
